# Prioritizing Sites for Protection and Restoration for Grizzly Bears (*Ursus arctos*) in Southwestern Alberta, Canada

**DOI:** 10.1371/journal.pone.0132501

**Published:** 2015-07-13

**Authors:** Andrew C. R. Braid, Scott E. Nielsen

**Affiliations:** Department of Renewable Resources, University of Alberta, Edmonton, Alberta, Canada; U.S. Geological Survey, UNITED STATES

## Abstract

As the influence of human activities on natural systems continues to expand, there is a growing need to prioritize not only pristine sites for protection, but also degraded sites for restoration. We present an approach for simultaneously prioritizing sites for protection and restoration that considers landscape patterns for a threatened population of grizzly bears (*Ursus arctos*) in southwestern Alberta, Canada. We considered tradeoffs between bottom-up (food resource supply) and top-down (mortality risk from roads) factors affecting seasonal habitat quality for bears. Simulated annealing was used to prioritize source-like sites (high habitat productivity, low mortality risk) for protection, as well as sink-like sites (high habitat productivity, high mortality risk) for restoration. Priority source-like habitats identified key conservation areas where future developments should be limited, whereas priority sink-like habitats identified key areas for mitigating road-related mortality risk with access management. Systematic conservation planning methods can be used to complement traditional habitat-based methods for individual focal species by identifying habitats where conservation actions (both protection and restoration) have the highest potential utility.

## Introduction

Applied conservation biology aims to protect undisturbed sites from future degradation, and to restore degraded sites to their former states. For many landscapes, the spectrum of site conditions ranges from pristine to destroyed [1]. More pristine sites could benefit from future protection, whereas degraded sites require restoration. However, needs for protection and restoration often outstrip the resources available to address them [2]. Conservation actions must therefore be prioritized [3]. Methods for prioritizing conservation actions frequently fall under the banner of systematic conservation planning, which identifies conservation goals or objectives and optimizes management actions to achieve them [4–6]. Although systematic conservation planning has frequently been used to optimize the design of protected area networks, there remains a need to expand these concepts to a wider area of conservation objectives and management actions, including landscape prioritization of sites for restoration [1,6]. The broadening scope of systematic conservation planning reflects the need for prioritization in all facets of applied conservation biology to encompass the full spectrum of site conditions.

Focal species are frequently used for land use and conservation planning because complete inventories of biodiversity are generally not practical [3,7,8]. They are typically well-studied, charismatic megafauna (flagship species) that often have large area requirements for maintaining viable populations, and are therefore thought to confer umbrella effects to other co-occurring species [7,9,10]. In some cases, focal species may also be considered keystone species if their role in ecosystem functioning is disproportionate relative to their abundance [6,8]. Traditionally, single-species conservation planning methods have relied mostly on spatially-explicit species habitat models (i.e., resource selection functions, species distribution models, and ecological niche models) to predict spatial distributions of species and in some cases to prioritize sites for conservation [11,12]. Examples include conservation planning for Amur tigers (*Panthera tigris altaica*; [13]), African elephants (*Loxodonta Africana*; [14]), and grizzly bears (*Ursus arctos*; [15]).

Habitat-based approaches to defining species habitat are sometimes replaced by analytical techniques that synthesize information about both habitat and population demographics. These methods acknowledge the need to use estimates of realized habitat quality (i.e., potential habitat quality balanced by information about survival or mortality risk; [16]) as the basis for effective conservation planning. Spatial population viability analyses (SPVAs) incorporate demographic and habitat data to predict species decline or recovery and are frequently used in conservation planning [7,17]. Two-dimensional approaches that explicitly consider trade-offs between bottom-up and top-down regulators of populations have also been used to estimate realized habitat quality and to identify areas where habitat restoration efforts are most likely to succeed [16,18–20].

Two major gaps with using these approaches in conservation planning still remain. First, they generally do not consider landscape context of individual sites, which can undermine the ecological relevance of their outputs [21]. And second, they seldom provide explicit prioritizations of sites for the two primary conservation tools of protection and restoration, thereby limiting their impetus for focusing management actions. Here we present an approach for simultaneously prioritizing sites for protection and restoration in the context of landscape patterns. This process is illustrated for a threatened population of grizzly bears in southwestern Alberta, Canada, by balancing seasonal habitats where bears forage against proximity to roads, which are tied to mortality risk [22–24]. More specifically, our objectives were to: (1) develop habitat quality indices that consider bottom-up factors (predicted distributions of important food resources) as well as top-down population regulators (road-based mortality risk); and (2) prioritize late-season source-like habitats (highly productive, low risk) for protection, and late-season sink-like habitats (highly productive, high risk) for restoration (access management), while considering the landscape context of bear habitat.

## Methods

### Ethics Statement

All field activities were conducted on public land and no endangered or protected species were involved in sampling work. As such, no specific permissions were required.

### Study Area

**Fig 1:**
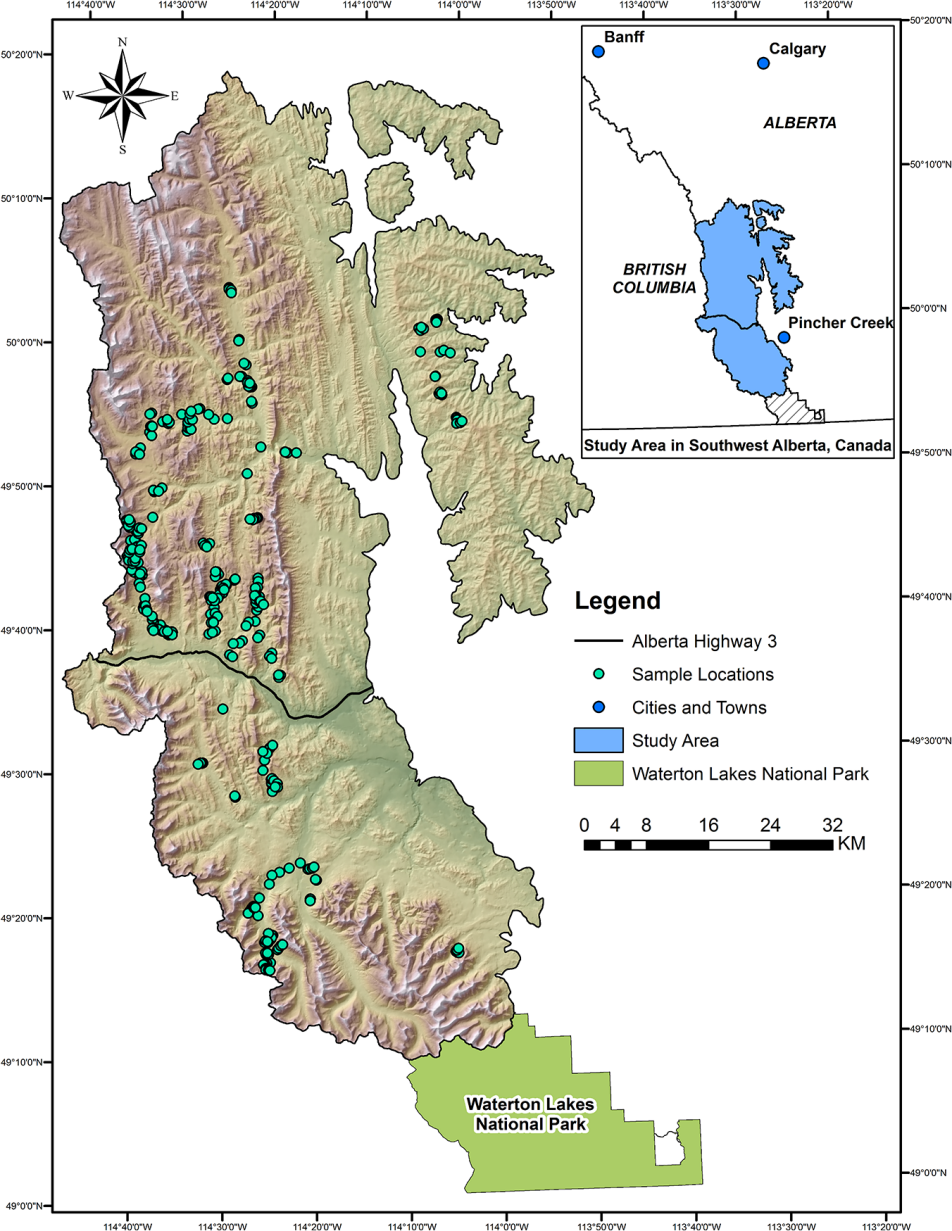
Location of study area in southwestern Alberta, Canada, with field plots indicated.

We sampled bear foods across a 5,065 km^2^ study area in southwestern Alberta (Fig 1) extending east from the British Columbia border to the edge of the foothills, and north approximately 125 km from the Waterton Lakes National Park boundary. The study area is characterized by mountains, high, rolling foothills, and deeply-cut glacial valleys [25]. Elevations in the study area range from 1155 m to 3009 m, with a mean elevation of 1672 m. Summers are short and cool (623 growing degree days > 5°C, mean annual temperature of -0.4°C), and mean annual precipitation is 798 mm [25]. Highly variable topography and geography yield a wide variety of plant communities. In general, open *Picea engelmannii* (Englemann spruce) and *Abies lasiocarpa* (subalpine fir) stands and herbaceous meadows occur at the highest elevations, whereas closed *Pinus contorta* (lodgepole pine) stands with *P*. *engelmannii* and *A*. *lasiocarpa* occur at moderate elevations [25,26]. Grasslands, mixed-wood forests, and open forests comprised of *Pseudotsuga menzeisii* (Douglas fir), *P*. *contorta*, and *Picea glauca* (white spruce) occur at lower elevations [25]. Although regeneration is relatively slow, timber harvesting is common to the area, especially north of Highway 3 [25,27]. Recreational use is prevalent, with the exception of the easternmost portion of the study area where landowners control access [27,28]. Parks and protected areas cover 414.2 km^2^ (8.2%) of the study area, primarily in the form of wildland provincial parks, natural areas, heritage rangelands, and ecological reserves. Access in these types of protected areas is predominantly limited to trails, and other forms of infrastructure and facilities are rare. A large portion of the study area (3402.0 km^2^, 67.1%) falls within the Rocky Mountains Forest Reserve, which is publically owned and is managed for timber production, fish and wildlife, recreation, energy development, and watershed maintenance. The remaining 1671.1 km^2^ (32.9%) falls within Alberta’s white zone, the majority of which is privately owned and is used primarily for grazing and agriculture.

### Focal Species Defining Grizzly Bear Habitat

Thirteen fruiting species were selected based on their prevalence in the study area (present at more than 10% of sample locations) and their known importance to regional grizzly bear diets [18,29–35]. Species included *Shepherdia canadensis* (Canada buffaloberry), *Vaccinium membranceum* (mountain huckleberry), *Amelanchier alinifolia* (saskatoon), *Ribes* spp. (gooseberry), *Arctostaphylos uva-ursi* (bearberry), *Lonicera involucrata* (black twinberry), *Sambucus racemosa* (black elderberry), *Fragaria virginiana* (wild strawberry), *Rubus idaeus* (wild red raspberry), *Rubus parviflorus* (thimbleberry), *Vaccinium caespitosum* (dwarf blueberry), *Vaccinium scoparium* (grouse whortleberry), and *Vaccinium myrtillus* (bilberry). *S*. *canadensis* and *V*. *membranaceum* typically dominate grizzly bear diets in Alberta and interior British Columbia during hyperphagia, a period between late summer and early fall when bears intensify foraging efforts to build body fat reserves for hibernation [22,29–35]. *A*. *alnifolia* has been identified as a significant component of grizzly bear diets in southwest Alberta between late July (lower elevations) and late October (higher elevations), while *A*. *uva-ursi* is targeted by bears during late spring (early May to mid-June) and early fall (early October to mid-November) [29]. *Ribes* spp., *S*. *racemosa*, and *L*. *involucrata* are utilized less frequently [18,29,31], but have the potential to produce significant amounts of fruit. The remaining species occur only to a limited extent in grizzly bear diets [18,31]; as such, their consumption is considered incidental to that of other more productive and nutritious species. Species were grouped into four categories of importance based on their prevalence in grizzly bear dietary scat analyses (Table 1). Dietary weights were assigned to each importance category based on the presence and importance of each species in regional grizzly bear diets using previously published scat-diet studies (Table 1; [18,29–35]).

**Table 1:** Focal species defining grizzly bear habitat.

Species name	Species code	Importance category	Dietary weight and optimization target
*Vaccinium membranaceum*	VMEM	Critical	0.300
*Shepherdia canadensis*	SCAN	Critical	0.300
*Amelanchier alnifolia*	AALN	Major	0.150
*Arctostaphylos uva-ursi*	AUVA	Major	0.150
*Ribes* spp. (Gooseberry)	RGB	Moderate	0.100
*Lonicera involucrata*	LINV	Moderate	0.100
*Sambucus racemosa*	SRAC	Moderate	0.100
*Rubus parvifolorus*	RPAR	Minor	0.025
*Rubus idaeus*	RIDA	Minor	0.025
*Vaccinium myrtillus*	VMUS	Minor	0.025
*Vaccinium caespitosum*	VCAE	Minor	0.025
*Vaccinium scoparium*	VSCO	Minor	0.025
*Fragaria virginiana*	FVIR	Minor	0.025

Categories of fruiting species importance based on prevalence in grizzly bear dietary scat analyses, and associated weights (used to generate an index of late-season habitat productivity, *H*_*LS*_) and conservation feature targets for Marxan optimization.

### Field Plots and Data Collection

Presence/absence data of grizzly bear foods were collected from 322 stratified field plots in southwestern Alberta (Fig 1) in 2012 (early July to mid-August) and 2013 (late May to mid-August) to characterize bottom-up resources with an emphasis on fruiting species. Plots were selected based on a stratification of Alberta Vegetation Inventory (AVI; [36]) classes and 100 m elevation zones (strata) using a geographic information system (GIS; [37]). Plots were placed at centroids of AVI polygons and chosen to be representative of environments in the region, while still being accessible (i.e., within 2.5 km of roads and trails). Sampling effort in each 100-m elevation zone (ranging from 1300 m to 2300 m) was weighted based on the frequency of available elevations in the study area. At each site, presence of fruit-producing species known to occur in regional grizzly bear diets, along with their respective reproductive stages (phenophases), were recorded along a 50-m belt transect with a belt width of 10 m (total plot size of 0.05 ha).

### Attractive Sink and Safe Harbour Habitats

Species distribution models were developed for each focal species using a purpose-built modeling approach with logistic regression (S1 File). Using this approach, an analyst makes variable selection decisions at each step of the modeling process. A suite of climate, landcover, terrain, and stand variables were considered during model building (Table A in S1 File). Presence of reproductive structures (flowering or fruiting) was then modeled (S1 File), again using logistic regression (0 –present, but no sign of reproduction; 1 –present with signs of reproduction), for each fruiting species to define fruiting habitat that would be relevant to bears during hyperphagia, which coincides with the period between late summer and early fall (“late-season”) when fruit ripens and is consumed by bears. Model estimates were used to create binary rasters (for both presence and fruiting models) for each species in a GIS [37]. Because the fruiting model was conditional on presence of the species, binary fruiting rasters were multiplied by the binary presence rasters for each species to produce binary rasters of fruiting given presence. These rasters were then summed across the study area using additive dietary weights (Table 1) to generate an index of late-season habitat productivity:

$$H_{LS}=\left[0.30\left(SCAN+VMEM\right)\right]+\left[0.15\left(AALN+AUVA\right)\right]+\left[0.10\left(RGB+LINV+SRAC\right)\right]+[0.05(FVIR+RPAR+RIDA+VSCO+VCAE+VMUS)]$$

where *H*_*LS*_ represented late-season habitat productivity within any given study area pixel (30-m resolution) with each four-letter species code corresponding to one of the thirteen focal fruiting species (Table 1). A road-based mortality risk index (*M*_*R*_) was calculated using a distance-to-access coefficient from a human-caused grizzly bear mortality risk model by Nielsen et al. [24]:

$$M_{R}=\text{exp}\left(-1.63d\right)\;/\;\left[1+exp\left(-1.63d\right)\right]$$

where *d* was distance in km to the nearest road. Both indices were rescaled to range from 0 to 1. Given the importance of food resources (particularly fruiting species) to grizzly bears during hyperphagia, *H*_*LS*_ was used to represent the bottom-up dimension within a two-dimensional habitat framework. *M*_*R*_ was used to represent the top-down dimension, and using a procedure similar to that of Nielsen et al. [20], attractive sink and safe harbour indices were estimated for the study area. Attractive sinks (also referred to as ecological traps) are areas where both habitat productivity and mortality risk are high, whereas safe harbours (source habitats) are areas where habitat productivity is high and mortality risk is low [20]. Thus, we defined our attractive sink (*AS*) and safe harbour (*SH*) indices as:

$$AS=H_{LS}\;\times \;M_{R}$$

and

$$SH=\;H_{LS}\;\times \;(1-M_{R})$$

where *AS* is an index of a site’s potential to be an attractive sink (0 = low, 1 = high), and *SH* is an index of a site’s potential to be a safe harbour (0 = low, 1 = high). These two habitat conditions were assumed to correlate with survival and reproduction (which is closely tied to nutritional state; [19]), both of which are responsible for regulating population growth [38]. Knowledge of the spatial distribution of these indices can aid conservation efforts by providing a basis for management actions directed at bolstering grizzly bear populations by mitigating mortality risk and/or fostering reproduction. In spite of this, representations of these indices across large areas can be difficult to translate into management action, creating a need to prioritize sites.

### Optimizing Sites using Marxan

Marxan is a spatially-explicit software tool developed to aid in the design of reserve systems (protected areas), and is commonly used to provide decision support for conservation planning [39–43]. Optimization using Marxan requires the definition of planning units–spatial units that summarize conservation features and costs (the “cost” of planning units is flexible and is not limited to economic measures). Marxan optimization algorithms include simulated annealing to identify many near-optimal sets (runs) of planning units that attempt to meet pre-defined conservation targets while minimizing associated costs [44]. Total cost of any given run is defined as the sum of planning unit costs, target penalties (which penalize solutions that do not meet conservation targets), and boundary costs (which penalize solutions with longer boundary lengths) [44]. We used hexagonal planning units to maximize the number of connections, which increases the effectiveness of manipulating the boundary length modifier (BLM). The BLM penalizes solutions with longer boundary lengths (i.e., less compactness), and thereby encourages the selection of planning units with shared boundaries. This reduces the overall fragmentation of solutions, which in turn yields more realistic options for conservation management [42,45]. The size of hexagonal planning units was set at 9 ha (shortest diagonal = 322.37 m) to maximize the total number of planning units while ensuring that their size was greater than the lowest resolution product that was used for modeling (300-m climate surfaces from Roberts et al. [46]). The value of conservation features (fruiting species) within each planning unit was defined as total pixels of habitat suitable for reproduction (from fruiting models) for each fruiting species within that planning unit. Conservation targets (proportions of the total amount of each conservation feature that must be included in each optimization solution) were set for each species using the same additive dietary weights (Table 1) that were used to calculate *H*_*LS*_ (late-season habitat productivity index). One output provided by Marxan is a summed solution, which summarizes the number of times each planning unit was selected across all runs. The summed solution is frequently used to quantify the relative irreplaceability of planning units [10,44,47]. Within the context of a given optimization framework, planning units selected in many runs likely have higher conservation value than planning units selected less frequently.

### Identifying Priority Sites for Protection and Restoration

To identify priority source-like habitats, we first ran an optimization in Marxan that used the mean road-based mortality index (*M*_*R*_) value of each planning unit as a cost. This tended to select for planning units away from roads (habitats with high *H*_*LS*_ values and low *M*_*R*_ values). Sink-like habitats were then identified by running an optimization that used the inverse of the mean road-based mortality index (*M*_*R*_ **)** value of each planning unit as a cost, which favoured the selection of planning units close to roads (habitats with high *H*_*LS*_ and *M*_*R*_ values). Using the sum of solutions from 100 iterations, priority source- and sink-like habitats were defined as any planning unit selected more than 50 times.

## Results

### Habitat Quality Indices

**Fig 2:**
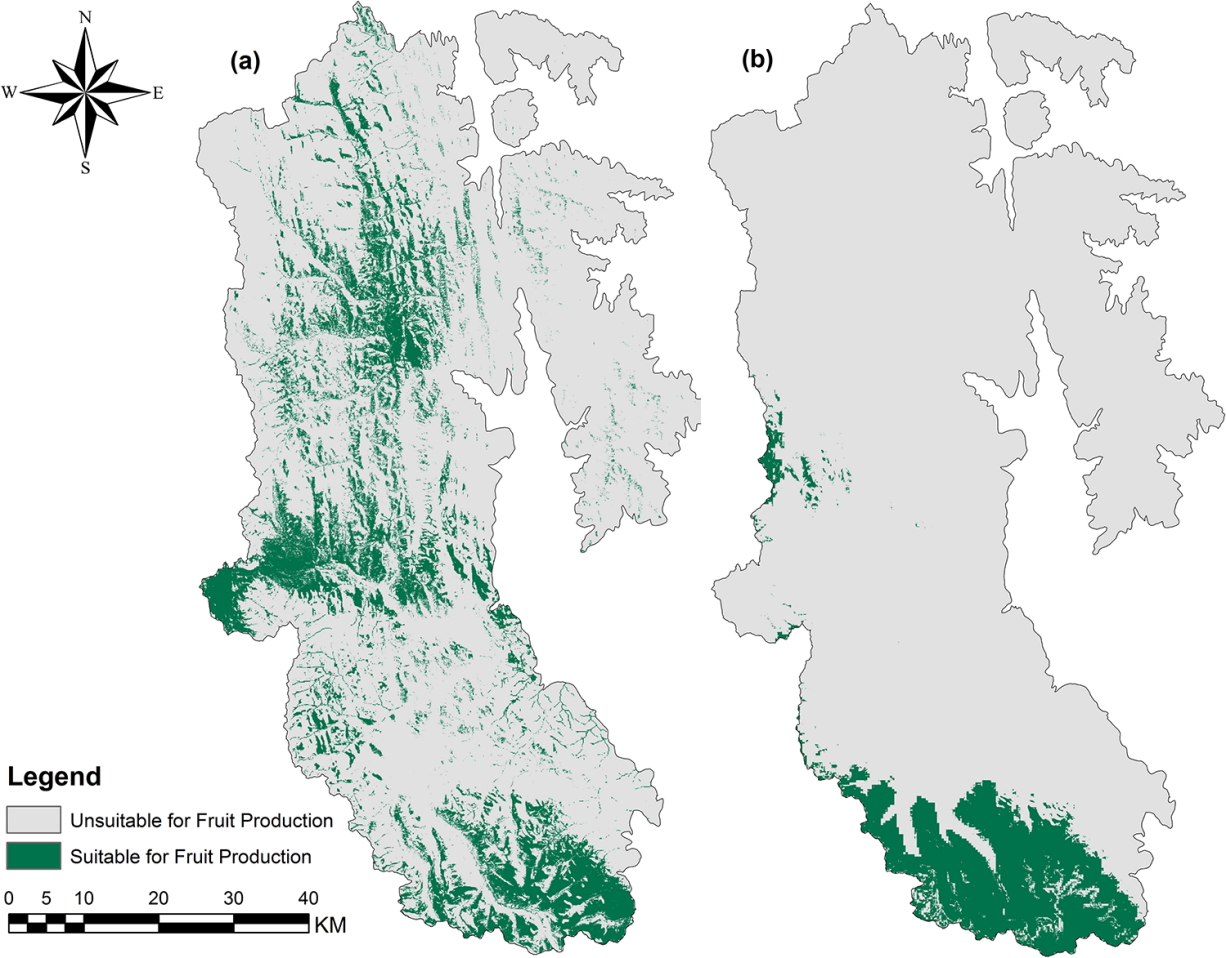
Binary fruiting maps for critical fruiting species: (a) *S*. *canadensis* and (b) *V*. *membranaceum*.

**Fig 3:**
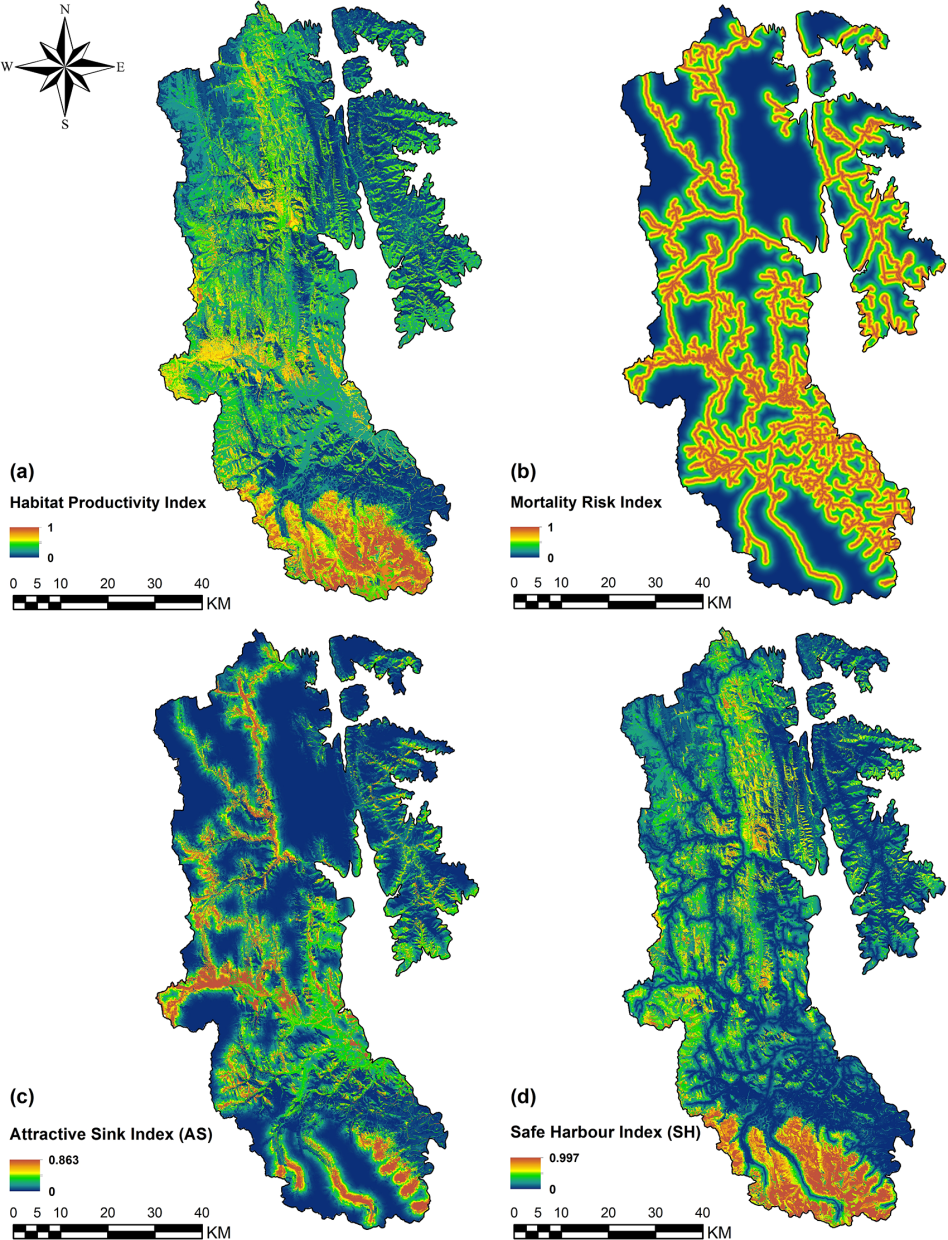
Maps of (a) late-season habitat productivity index (*H*_*LS*_); (b) road-based mortality risk index (*M*_*R*_); (c) attractive sink index (*AS*); and (d) safe harbour index (*SH*).

Late-season habitat productivity (*H*_*LS*_) values were highest where there was considerable overlap of fruiting species (particularly critical species, *S*. *canadensis* and *V*. *membranaceum*) habitat (Fig 2, Fig 3A). Mortality risk (*M*_*R*_) values were highest on or very near to roads (M_R_ ≈ 1.0), but dropped to 0.61 and 0.33 at distances of 500 m and 1000 m from roads, respectively (Fig 3B). Attractive sink (*AS*) index values were highest where both *H*_*LS*_ and *M*_*R*_ values were high (i.e. productive fruiting habitats close to roads), whereas safe harbour (*SH*) index values were highest in areas with high *H*_*LS*_ values and low *M*_*R*_ values (i.e. productive fruiting habitats away from roads; Fig 3C and Fig 3D, respectively).

### Priority Sites for Protection and Restoration

**Fig 4:**
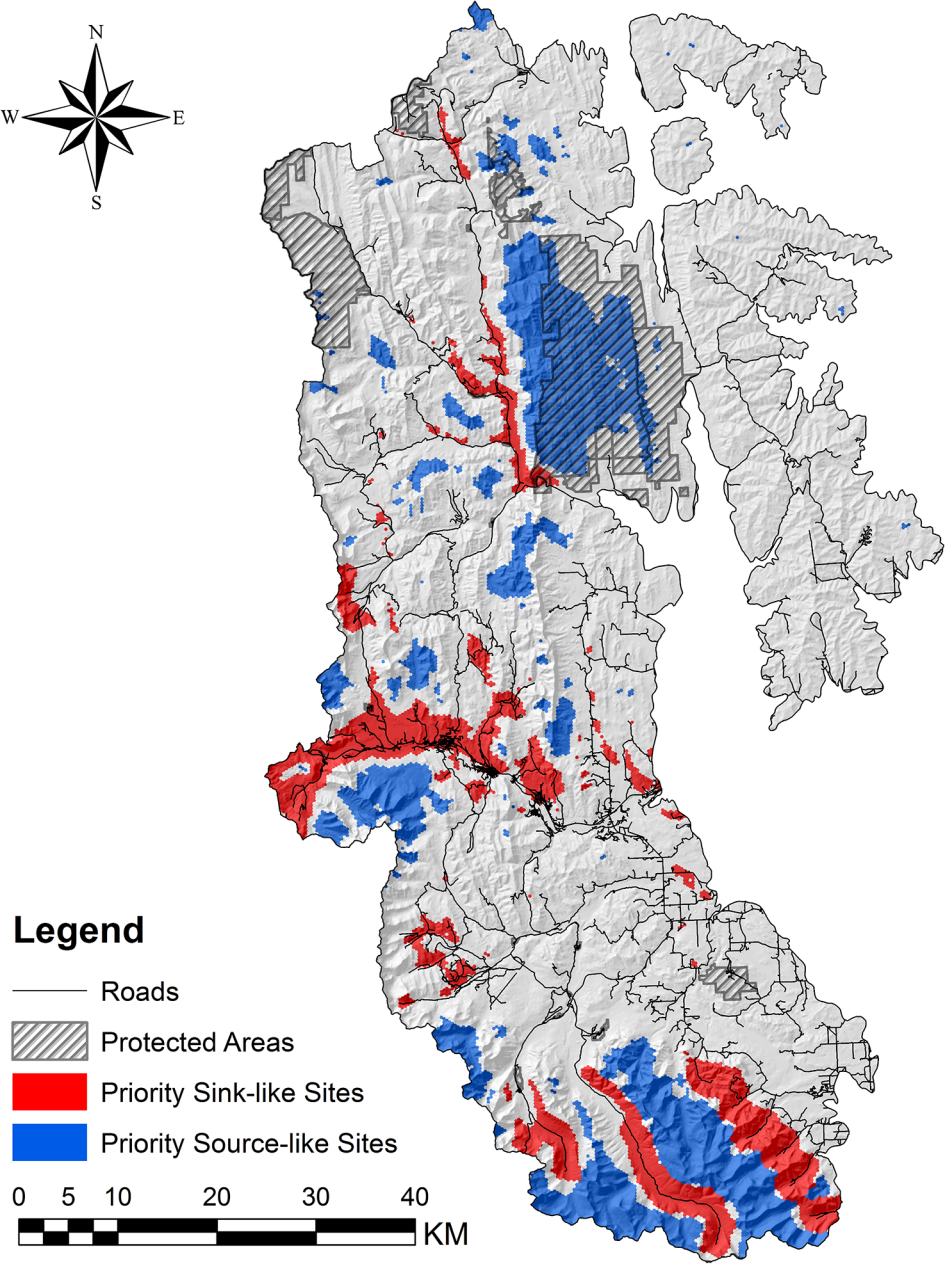
Map showing priority source- and sink-like sites in the southwestern Alberta study area.

Optimization analysis in Marxan identified 425.8 km^2^ of priority sink-like habitat (Fig 4). Of this, 62.7% was attributable to unimproved roads and truck trails (42.8% and 19.9% respectively). Paved roads accounted for only 6.1% of priority sink-like planning units, whereas gravel roads accounted for 26.0% of sink-like planning units. Mean distance to road for priority sink-like habitats was 341.4 m (SE = 5.3 m). A total of 656.9 km^2^ of priority source-like habitat was identified, 24.7% (162.5 km^2^) of which is currently protected (Fig 4). Of this overlap between priority source-like habitats and current protected areas, 97.8% (158.8 km^2^) occurred in two adjacent existing protected areas. Patch sizes for source-like habitats ranged from 0.08 km^2^ to 219.6 km^2^ (mean patch size 8.01 km^2^; SE = 3.37 km^2^). Mean distance to road for source-like habitats was 3.54 km (SE = 0.017 km), and minimum distance to road was 957 m.

## Discussion

### Habitat Quality Indices

Mortality risk index (*M*_*R*_) values were comparable to empirical data from previous studies that examined human-caused grizzly bear mortalities in relation to roads. Benn and Herrero [23] found that all human-caused mortalities between 1971 and 1998 with accurate locations in Banff and Yoho National Parks occurred within 500 m of roads or 200 m of trails. Aune and Kasworm [48] found that 63% of human-caused mortalities occurred within 1000 m of roads. Finally, Schwartz et al. [49] found a strong positive relationship between “secure” habitat (habitat patches greater than 4.05 ha and more than 500 m from a road) and grizzly bear survival in the Greater Yellowstone Ecosystem between 1983 and 2003. The mortality risk index remained high (> 0.61) within 500 m of roads, moderate (> 0.33) within 1000 m of roads, and dropped substantially thereafter. The index of attractive sink habitat was highest where fruiting habitats overlapped for multiple important fruiting species in areas close to roads. Conversely, the index of safe harbour habitat was highest where fruiting species habitats coincided in areas away from roads. These habitat indices provide a measure of the spatial interaction between top-down and bottom-up population factors and can be used to quantify the risk or security of grizzly bear habitat during hyperphagia. Even so, they have limited applicability at a management level unless they are complemented by specific criteria for management action. For example, Nielsen et al. [20] applied thresholds to habitat quality indices to define relative habitat states, and specifically recommended management strategies, such as protection and restoration. However, even when habitat states are clearly defined, managing all habitats across a large region is not feasible given that conservation resources are limited. There is a need to therefore prioritize sites to provide a stronger basis for focusing management actions.

### Priority Sites for Protection and Restoration

Optimization using Marxan identified priority late-season source- and sink-like sites for protection and restoration. These sites (planning units) were selected in the majority of runs and represented the most valuable habitats for meeting pre-defined conservation targets (focal fruiting species presence) while minimizing costs (mortality risk associated with roads). Overall, the co-occurrence of both critical fruiting species, or one critical fruiting species and several major or moderate fruiting species, determined the selection of priority sites for conservation (either away from roads for source-like sites, or close to roads for sink-like sites). The mean distance-to-road for priority sink-like sites of 341.4 m was consistent with observed patterns of most human-caused grizzly bear mortalities occurring in close proximity to roads (i.e., within 500 m) [23, 49–51]. Similarly, minimum distance-to-road for priority source-like sites was 971 m, which is outside the “high risk” zone for grizzly bears [23,49].

Mean patch size of priority source-like habitats was 8.01 km^2^, and ranged from 0.08 km^2^ to 219.6 km^2^. The minimum patch size of approximately 8 ha is still relevant to foraging grizzly bears (i.e., large enough to contain a significant quantity of fruit), but is too small to justify individual protection or restoration actions. Larger patches are indicative of Marxan grouping highly valuable planning units that occur in close proximity, including planning units with less value (i.e., conservation features) to establish connectivity between high-value sites. The amount of overlap between protected areas and priority source-like sites indicates that a large portion of important late-season grizzly bear foraging habitat is not currently protected from future road access. The majority of this overlap (97.8%) occurs in two adjacent protected areas, suggesting that current protection of priority source-like sites is also geographically skewed and possibly lacking in other parts of the study area. Only 13.0% of the study area was identified as priority source-like habitat, which highlights the importance of these sites to grizzly bear habitat management in the region. The majority of priority source-like sites that are currently protected occur in a wildland provincial parks, heritage rangelands, and natural areas where road access is limited (in the case of wildland provincial parks and heritage rangelands) or non-existent (in the case of natural areas). Future protection of priority source-like sites that are currently unprotected must include restrictions on road development to maintain their security and effectiveness. New access features will also modify the “costs” (road-based mortality risk) associated with planning units if they are closer than existing roads, and may shift subsequent optimization solutions. Where road installation in close proximity to priority source-like sites is necessary for industrial activity, all access points should be decommissioned following resource extraction. Restoration should follow to discourage people from accessing these areas and to maintain the security of nearby priority source-like sites.

Most grizzly bear mortalities occur on or near roads where public access is permitted [52]. The majority of priority sink-like sites were associated with unimproved roads or truck trails, which are low-volume roads that are used almost exclusively for recreational purposes and represent the best candidates for permanent closure and restoration. Almost all remaining priority sink-like sites (26.0%) were attributable to gravel roads, which generally see more frequent use from both industry and the public and require significant monetary investments for construction and maintenance. Permanent closure and restoration of gravel roads is often an unpopular management option with some interest groups. Instead, stakeholders may be more amenable to modified access strategies such as seasonal closures during hyperphagia or gated access to restrict public use of industry roads. Limiting public access could lead to significant reductions of mortality risk; however, relaxing access restrictions following the completion of industrial activities would require careful consideration since grizzly bears can become habituated to industrial activity on roads [52–54].

### Prioritization and Landscape Patterns in Conservation Planning

Conservation planning approaches using focal species rarely explicitly prioritize sites for both protection and restoration, in spite of the growing need to not only maximize the efficiency of management efforts, but to also target degraded sites for restoration. Definitions of habitat quality or relative habitat states can be complemented with prioritizations of candidate sites for conservation to provide additional impetus for management action. Prioritization methods such as the one employed in this study can be used with measures of habitat quality (i.e., habitat indices) to identifying habitats where the potential utility of conservation actions (both protection and restoration) is highest. A common objective of systematic conservation planning is to design minimum-cost solutions to meet quantitative conservation goals [2,43]. As demonstrated here, Marxan achieves this by identifying portfolios of sites (planning units) that have the highest value for meeting conservation feature targets at the lowest “cost”‘.

Furthermore, conservation planning approaches that employ the use of a focal species frequently ignore landscape patterns [21]. Particularly for species with large ranges, the quality of a site is dictated not only by bottom-up and top-down factors, but also by the quality of nearby or connected sites [55]. Thus, management actions based on fine-scale definitions of habitat quality that do not incorporate the surrounding landscape context may lack ecological relevance. An isolated high quality site may have less ecological value than a group of connected moderate quality sites [55,56]. Similarly, low quality sites that join groupings of high quality sites together may have increased ecological value because they promote habitat connectivity [55,56]. Connectivity of sites is also appealing from an operational standpoint, because management of dispersed blocks of habitat may be logistically impractical [42,46]. Marxan incorporates such landscape patterns into prioritization solutions via its boundary length modifier, which encourages the selection of connected or adjacent planning units [46]. While this increases the number of planning units required to meet conservation targets, it promotes the connectivity of solutions, making them more ecologically relevant and yielding more realistic management options [46].

## Conclusions

Systematic conservation planning identifies conservation goals and optimizes management actions to achieve them, but has been used mostly for optimizing the design of protected area networks. The need to also restore sites, though already apparent, will only grow as human environmental impacts continue to intensify. Systematic conservation planning provides an avenue for maximizing the utility of limited conservation resources, but its scope must be expanded to encompass the full spectrum of site conditions. For degraded sites, the aim of restoration efforts must be to mitigate the “costs” associated with them, as this will ultimately drive their selection during prioritization. Similarly, the focus of management strategies for sites prioritized for protection should be to proactively restrict increases in “cost”‘. The optimization method we present here simultaneously prioritizes sites for protection and restoration, addressing the need to protect undisturbed sites from degradation, as well as the need to restore degraded sites to their former states. Systematic approaches to focal species conservation planning can form the basis for targeted management actions, and should be combined with habitat indices (overall measures of regional habitat quality) to provide context for management decisions.

## Supporting Information

S1 File Modeling species distributions to define grizzly bear habitat. (DOCX)
