# Supplementary material for: Prioritizing Sites for Protection and Restoration for Grizzly Bears (Ursus arctos) in Southwestern Alberta, Canada
Source: PLoS One. 2015 Jul 13;10(7):e0132501. doi: 10.1371/journal.pone.0132501 (PMC4500459; doi:10.1371/journal.pone.0132501)
Supplement: S1 File — (DOCX) [file pone.0132501.s001.docx]

# Modeling Species Distributions to Define Grizzly Bear Habitat

Species distributions and predicted presence of reproductive structures were modeled using a purpose-built modeling approach. This approach began with univariate logistic regression analysis (0 – absence; 1 – presence) of each covariate (Table A). Covariates were then ranked for importance using Akaike information criterion (AIC), and models were built by first including the top-ranked covariate, and then successively adding the next-highest ranked covariate. Highly correlated variables (r > |0.7|) were not included in the same model. If a covariate was added to the model and did not have a significant p-value (i.e., p < 0.1), it was discarded. This process was iterated until all covariates had been considered. Quadratic forms of each climate covariate and selected terrain variables were also included in the model building process to test for possible non-linear relationships. Following completion of a main effects model, interaction terms were considered on the basis of hypothesized relationships and statistical significance. Model complexity was limited to no more than one variable per 6 observations (Table B, Table C). Finally, model fit was assessed using the receiver operating characteristic (ROC) area under the curve (AUC), as well as a Hosmer-Lemeshow goodness-of-fit test using 10 groups (Table B, Table C). Previous work for these species using similar predictor variables with independent data has shown similar accuracy [1-3]. See also Nielsen et al. 2010 [4] for species distribution models with similar environmental relationships for these species with reported ROC AUC scores.

For fruiting models, only field plots where a particular species was observed were included, and observations were restricted to the period where reproductive structures were observed for that species. This allowed separation of sites where the species may be present but not fruiting (such as presence under tree cover). Optimal probability cut-off values were estimated for each species using equalized sensitivity-specificity probability thresholds (i.e., intersection of sensitivity and specificity curves) from ROC calculations [5-6]. These values were used to reclassify original probabilities and generate binary raster layers of predicted presence-absence (for both presence and fruiting models) across the study area (0 – absence; 1 – presence). Binary fruiting rasters were multiplied by binary presence rasters for each species to produce binary rasters of fruiting given presence.

## Species Distributions

Predicted presence of fruiting species was influenced mainly by temperature (climate) and terrain-derived variables, while stand (cut block and canopy cover) and landcover variables were generally less important (Table D). Fruiting models, on the other hand, were driven mainly by climate (both temperature- and precipitation-based climate normals), terrain-derived, and stand-level variables (Table E). For instance, canopy cover was important for predicting fruiting for seven focal species, with moderate levels of canopy cover favouring fruiting for *S. canadensis*, *L. involucrata*, and *F. virginiana*, and low levels of canopy cover favouring fruiting for *A. uva-ursi*, *R. parviflorus*, *V. myrtillus*, and *V. caespitosum*.

## Environmental Responses for Critical Fruiting Species

Predicted presence of *S. canadensis* was positively related to moderate frost-free periods, as well as areas of low canopy cover, high soil wetness (cti), and low heat load values (Table D). Interaction effects between canopy cover, soil wetness, and heat load values suggest that *S. canadensis* is more likely to occur when two or more of these conditions are satisfied (i.e. sites with low canopy cover and high soil wetness). *V. membranaceum* was predicted to occur in areas with higher canopy cover, lower heat load index values, and areas that receive more precipitation as snow. *V. membranaceum* occurrence was also predicted to be lower at moderate January minimum temperatures (tmn01). Contrary to its relationship with canopy cover (positive), *V. membranaceum* presence was also associated with cut blocks. Finally, an interaction effect between canopy cover and heat load values indicated that *V. membranceum* was more likely to occur on sites with lower heat loads and lower canopy cover. *S. canadensis* fruiting was negatively associated with moderate January minimum temperatures (tmn01), but positively associated with higher heat load values (Table E). *V. membranaceum* fruiting was not significantly related to any terrain-derived or stand-level variables, but instead, was strongly predicted by a summer heat-to-moisture index.

## References

1. Nielsen SE, Boyce MS, Stenhouse GB, Munro RHM. Development and testing of phenologically driven grizzly bear habitat models. Ecoscience. 2003;10:1-10.
2. Roberts DR, Nielsen SE, Stenhouse GB. Idiosyncratic responses of grizzly bear habitat to climate change based on projected food resource changes. Ecol Appl. 2014;24:1144-1154.
3. Nielsen SE, Munro RHM, Bainbridge EL, Stenhouse GB, Boyce MS. Grizzly bears and forestry II. Distribution of grizzly bear foods in clearcuts of west-central Alberta, Canada. Forest Ecol Manag. 2004;199:67-82.
4. Nielsen SE, McDermid G, Stenhouse GB, Boyce MS. Dynamic wildlife habitat models: Seasonal foods and mortality risk predict occupancy-abundance and habitat selection by grizzly bears. Biol Cons. 2010;143:1623-1634.
5. Pearce J, Ferrier S. Evaluating the predictive performance of habitat models developed using logistic regression. Ecol Model. 2000;133:225-245.
6. Boyce MS, Vernier PR, Nielsen SE, Schmiegelow FKA. Evaluating resource selection functions. Ecol Model. 2002;157:281-300.
7. Wang T, Hamann A, Spittlehouse DL, Murdock TQ. ClimateWNA – High-Resolution Spatial Climate Data for Western North America. J Appl Meteor Climatol. 2012;51:16-29.
8. McDermid GJ, Franklin SE, LeDrew EF. Remote sensing for large-area habitat mapping. Prog Phys Geogr. 2005;29:449-474.

**Table A.** **Suite of covariates used to model distributions and habitats suitable for reproduction for thirteen fruiting species in southwestern Alberta.**

| **Category** | **Model Covariates** | **Code** | **Units** | **Source** |
| --- | --- | --- | --- | --- |
| *Climate* | Annual heat-to-moisture index | ahm | Unitless | Wang et al., 2012 [7]; Roberts et al., 2014 [2] |
|  | Beginning of frost-free period | bffp | Day of year | Wang et al., 2012 [7]; Roberts et al., 2014 [2] |
|  | Climatic moisture deficit | cmd | mm | Wang et al., 2012 [7]; Roberts et al., 2014 [2] |
|  | Climate moisture index | cmi | Unitless | Wang et al., 2012 [7]; Roberts et al., 2014 [2] |
|  | Climate moisture index (June, July, and August) | cmijja | Unitless | Wang et al., 2012 [7]; Roberts et al., 2014 [2] |
|  | Degree-days below 0 °C | dd0 | Degree Days | Wang et al., 2012 [7]; Roberts et al., 2014 [2] |
|  | Degree-days above 5 °C | dd5 | Degree Days | Wang et al., 2012 [7]; Roberts et al., 2014 [2] |
|  | Ending of frost-free period | effp | Day of year | Wang et al., 2012 [7]; Roberts et al., 2014 [2] |
|  | Est. extreme minimum temperature, 30-yr normal | emt | °C | Wang et al., 2012 [7]; Roberts et al., 2014 [2] |
|  | Reference atmospheric evaporative demand | eref | mm | Wang et al., 2012 [7]; Roberts et al., 2014 [2] |
|  | Frost-free period | ffp | Days | Wang et al., 2012 [7]; Roberts et al., 2014 [2] |
|  | Mean annual precipitation | map | mm | Wang et al., 2012 [7]; Roberts et al., 2014 [2] |
|  | Mean annual temperature | mat | °C | Wang et al., 2012 [7]; Roberts et al., 2014 [2] |
|  | Mean coldest-month temperature | mcmt | °C | Wang et al., 2012 [7]; Roberts et al., 2014 [2] |
|  | Mean May-to-September precipitation | msp | mm | Wang et al., 2012 [7]; Roberts et al., 2014 [2] |
|  | Mean warmest-month temperature | mwmt | °C | Wang et al., 2012 [7]; Roberts et al., 2014 [2] |
|  | Number of frost-free days | nffd | Unitless | Wang et al., 2012 [7]; Roberts et al., 2014 [2] |
|  | Precipitation as snow | pas | mm | Wang et al., 2012 [7]; Roberts et al., 2014 [2] |
|  | Summer precipitation | pptsm | mm | Wang et al., 2012 [7]; Roberts et al., 2014 [2] |
|  | Winter precipitation | pptwt | mm | Wang et al., 2012 [7]; Roberts et al., 2014 [2] |
|  | Summer heat-to-moisture index | shm | Unitless | Wang et al., 2012 [7]; Roberts et al., 2014 [2] |
|  | Average summer temperature | tavsm | °C | Wang et al., 2012 [7]; Roberts et al., 2014 [2] |
|  | Average winter temperature | tavwt | °C | Wang et al., 2012 [7]; Roberts et al., 2014 [2] |
|  | Continentality (MWMT – MCMT) | td | °C | Wang et al., 2012 [7]; Roberts et al., 2014 [2] |
|  | Minimum temperature in January | tmn01 | °C | Wang et al., 2012 [7]; Roberts et al., 2014 [2] |
|  | Maximum temperature in July | tmx07 | °C | Wang et al., 2012 [7]; Roberts et al., 2014 [2] |

**Table A (Continued).** **Suite of covariates used to model distributions and habitats suitable for reproduction for thirteen fruiting species in southwestern Alberta.**

| **Category** | **Model Covariates** | **Code** | **Units** | **Source** |
| --- | --- | --- | --- | --- |
| *Landcover* | Dense coniferous forest | dcf | Category | McDermid et al., 2005 [8] |
|  | Moderate coniferous forest | mcf | Category | McDermid et al., 2005 [8] |
|  | Open coniferous forest | ocf | Category | McDermid et al., 2005 [8] |
|  | Mixed forest | mxf | Category | McDermid et al., 2005 [8] |
|  | Broadleaf forest | blf | Category | McDermid et al., 2005 [8] |
|  | Shrub | shb | Category | McDermid et al., 2005 [8] |
|  | Herbaceous | hrb | Category | McDermid et al., 2005 [8] |
|  | Agriculture | agr | Category | McDermid et al., 2005 [8] |
|  | Barren | bar | Category | McDermid et al., 2005 [8] |
|  | Water | wat | Category | McDermid et al., 2005 [8] |
| *Terrain* | Compound topographic index | cti | Unitless | This study – derived from digital elevation model |
|  | Slope aspect index | sai | Unitless | This study – derived from digital elevation model |
|  | Potential direct incident radiation | pdir | Unitless | This study – derived from digital elevation model |
|  | Heat load index | hli | Unitless | This study – derived from digital elevation model |
| *Disturbance* | Cutblock binary | cb | Category | This study – derived from cutblock polygon layer |
| *Canopy* | Canopy cover | cc | % | McDermid et al., 2005 [8] |

**Table B. Selected statistics for presence models.**

| **Species** | **Prevalence** | ***K*** | **Model**  **Complexity** | **LL** | **AIC** | **Probability Threshold** | **ROC AUC** | **H-L GOF** |
| --- | --- | --- | --- | --- | --- | --- | --- | --- |
| *Vaccinium membranaceum* | 0.121 | 8 | 40.3 | -37.02 | 90.04 | 0.213 | 0.978 | 0.999 |
| *Shepherdia canadensis* | 0.416 | 10 | 32.2 | -183.40 | 386.81 | 0.424 | 0.758 | 0.228 |
| *Amelanchier alnifolia* | 0.382 | 6 | 53.7 | -154.53 | 321.06 | 0.414 | 0.834 | 0.333 |
| *Arctostaphylos uva-ursi* | 0.214 | 12 | 26.8 | -128.30 | 280.61 | 0.234 | 0.812 | 0.838 |
| *Ribes* spp. (Gooseberry) | 0.472 | 8 | 40.3 | -195.20 | 406.40 | 0.488 | 0.729 | 0.702 |
| *Lonicera involucrata* | 0.196 | 11 | 29.3 | -127.50 | 276.99 | 0.212 | 0.800 | 0.593 |
| *Sambucus racemosa* | 0.140 | 11 | 29.3 | -90.01 | 202.02 | 0.147 | 0.871 | 0.968 |
| *Rubus parvifolorus* | 0.335 | 4 | 80.5 | -156.29 | 320.57 | 0.291 | 0.802 | 0.073 |
| *Rubus idaeus* | 0.161 | 4 | 80.5 | -123.91 | 255.81 | 0.155 | 0.748 | 0.413 |
| *Vaccinium myrtillus* | 0.394 | 11 | 29.3 | -161.06 | 344.13 | 0.445 | 0.824 | 0.367 |
| *Vaccinium caespitosum* | 0.193 | 6 | 53.7 | -126.92 | 265.83 | 0.189 | 0.795 | 0.625 |
| *Vaccinium scoparium* | 0.239 | 6 | 53.7 | -130.69 | 273.37 | 0.228 | 0.843 | 0.432 |
| *Fragaria virginiana* | 0.721 | 13 | 24.8 | -144.98 | 315.96 | 0.696 | 0.814 | 0.599 |

Prevalence is the proportion of sites occupied (for presence models) or proportion of sites occupied with presence of reproductive structures (for fruiting models). *K* is the total number of model variables, including the constant. Model complexity is the total number of observations divided by the total number of variables, including the constant. LL is the value of the maximized log-likelihood equation. AIC is the value of the Akaike information criterion for a given model. Probability threshold refers to optimal probability cut-off values estimated using equalized sensitivity-specificity probability thresholds from receiver operating characteristic (ROC) calculations. ROC AUC refers to the area under the ROC curve, which provides a measures of a model’s predictive ability. H-L GOF refers to the Hosmer-Lemeshow goodness-of-fit test with 10 groups (probability > Hosmer-Lemeshow χ^2^).

**Table C. Selected statistics for fruiting models.**

| **Species** | **Prevalence** | ***K*** | **Model**  **Complexity** | **LL** | **AIC** | **Probability Threshold** | **ROC AUC** | **H-L GOF** |
| --- | --- | --- | --- | --- | --- | --- | --- | --- |
| *Vaccinium membranaceum* | 0.806 | 2 | 12.0 | -12.04 | 28.08 | 0.636 | 0.894 | 0.989 |
| *Shepherdia canadensis* | 0.556 | 7 | 19.0 | -73.74 | 161.49 | 0.565 | 0.777 | 0.518 |
| *Amelanchier alnifolia* | 0.487 | 7 | 17.0 | -51.18 | 116.36 | 0.405 | 0.855 | 0.490 |
| *Arctostaphylos uva-ursi* | 0.591 | 6 | 11.0 | -29.20 | 70.40 | 0.567 | 0.849 | 0.814 |
| *Ribes* spp. (Gooseberry) | 0.584 | 5 | 29.8 | -86.74 | 183.48 | 0.573 | 0.748 | 0.672 |
| *Lonicera involucrata* | 0.762 | 8 | 7.88 | -13.27 | 42.53 | 0.624 | 0.967 | 0.639 |
| *Sambucus racemosa* | 0.822 | 6 | 7.50 | -13.59 | 39.18 | 0.792 | 0.878 | 0.329 |
| *Rubus parvifolorus* | 0.752 | 8 | 12.6 | -35.73 | 87.47 | 0.778 | 0.893 | 0.118 |
| *Rubus idaeus* | 0.689 | 5 | 9.00 | -19.80 | 49.59 | 0.677 | 0.845 | 0.427 |
| *Vaccinium myrtillus* | 0.758 | 6 | 15.8 | -42.90 | 97.79 | 0.542 | 0.868 | 0.184 |
| *Vaccinium caespitosum* | 0.682 | 7 | 6.29 | -10.11 | 34.22 | 0.498 | 0.973 | 0.442 |
| *Vaccinium scoparium* | 0.707 | 6 | 12.5 | -27.53 | 67.06 | 0.760 | 0.892 | 0.630 |
| *Fragaria virginiana* | 0.420 | 10 | 23.1 | -127.61 | 275.22 | 0.409 | 0.774 | 0.136 |

Prevalence is the proportion of sites occupied (for presence models) or proportion of sites occupied with presence of reproductive structures (for fruiting models). *K* is the total number of model variables, including the constant. Model complexity is the total number of observations divided by the total number of variables, including the constant. LL is the value of the maximized log-likelihood equation. AIC is the value of the Akaike information criterion for a given model. Probability threshold refers to optimal probability cut-off values estimated using equalized sensitivity-specificity probability thresholds from receiver operating characteristic (ROC) calculations. ROC AUC refers to the area under the ROC curve, which provides a measures of a model’s predictive ability. H-L GOF refers to the Hosmer-Lemeshow goodness-of-fit test with 10 groups (probability > Hosmer-Lemeshow χ^2^).

**Table D. Logistic regression models predicting the occurrence of late-season grizzly bear food resources (fruiting species) in Southwestern Alberta.**

| **Species** | **Final Model** |
| --- | --- |
| *V. membranaceum* | 0.048*pas* – 1.3*hli* + 0.18*cc* + 2.3*cb* – 529*tmn01* – 18*tmn01*^2^ – 0.21(*hli* × *cc*) – 3929 |
| *S. canadensis* | –0.25*msp* + 0.00034*msp*^2^ + 19*effp* – 0.038*effp*^2^ – 0.044*cc* + 0.33*cti* – 3.4*hli* + 0.15(*hli* × *cc*) – 0.0051(*cti* × *cc*) – 2251 |
| *A. alnifolia* | –0.012*dd0* + 0.12*ahm* + 3.5*hli* – 3.1*sai* + 15*sai*^2^ + 9.4 |
| *A. uva-ursi* | –1.9*shm* + 0.023*shm*^2^ + 33*effp* – 0.066*effp*^2^ + 2.5*ocf* - 0.85*tmn01* + 63hli – 47*hli*^2^ – 1.6*bar* + 4.9*pdir* – 0.24*cti* – 4144 |
| *Ribes* spp. (Gooseberry) | 0.88*shm* – 0.013*shm*^2^ + 24*hli* – 19*hli*^2^ – 3.8*pdir* – 0.72*mcf* + 0.15*cti* – 21 |
| *L. involucrata* | 5.9*cb* – 2.9*pdir* + 0.063*pas* – 0.000075*pas*^2^ + 2.4*cti* – 0.11*cti*^2^ + 0.84*tmn01* + 1.6*ffp* – 0.010*ffp*^2^ – 7.3(*pdir* × *cb*) – 73 |
| *S. racemosa* | 6.2*ahm* – 0.23*ahm*^2^ – 0.031*cc* – 39*td* + 0.82*td*^2^ + 5.2*cb* – 5.9*pdir* + 0.13*cti* + 0.028*cb*^2^ – 0.63(*cti* × *cb*) + 423 |
| *R. parviflorus* | 1.2*emt* + 0.0050*map* + 0.50*tmx07* + 35 |
| *R. idaeus* | 0.26*effp* + 0.51*td* – 0.019*cc* – 77 |
| *V. myrtillus* | –0.17*cb* + 0.077*cmd* – 0.00057*cmd*^2^ + 0.082*mcmt* – 0.0064*map* + 3.0*sai* – 20*sai*^2^ – 36*td* + 0.77*td*^2^ + 5.3(*sai* × *cb*) + 432 |
| *V. caespitosum* | 31*effp* – 0.061*effp*^2^ – 1.2*tmn01* – 0.021*ppt_sm* – 2.3*sai* –3894 |
| *V. scoparium* | –0.47*effp* + 0.032*map* – 0.000020*map*^2^ + 1.7*bar* + 0.58*tmn01* + 111 |
| *F. virginiana* | 6.3*pdir* + 2.4*mxf* – 3.9*cb* + 0.20*cti* + 0.044*cc* – 0.00076*cc*^2^ + 27*hli* – 22*hli*^2^ – 0.042*pptwt* + 0.000083*pptwt*^2^ – 1.2*bar* + 8.2(*pdir* × *cb*) – 8.0 |

**Table E. Models for predicting suitable fruiting habitat for late-season grizzly bear food resources (fruiting species) in Southwestern Alberta.**

| **Species** | **Final Model** |
| --- | --- |
| *V. membranaceum* | –0.68shm + 26 |
| *S. canadensis* | 0.063*cc* – 0.0012*cc*^2^ + 56*tmn01* + 1.8*tmn01*^2^ + 6.7*hli* + 0.20*bffp* + 399 |
| *A. alnifolia* | –7.1*cti* + 0.39*cti*^2^ + 8.2*pdir* – 0.67*cb* – 0.040*cc* + 0.030(*cb* × *cc)* + 25 |
| *A. uva-ursi* | –3.0*ppt_sm* + 0.0078*ppt_sm*^2^ + 15*pdir* + 0.16*mcmt* – 0.025*cc* + 280 |
| *Ribes* spp. (Gooseberry) | –0.76*tmx07* + 6.8*cb* + 3.5*hli* – 12(*hli* × *cb*) + 14 |
| *L. involucrata* | 7.2*cb* + 0.86*cc* – 0.013*cc*^2^ – 263*tmn01* – 8.6*tmn01*^2^ + 5.0*dcf* + 3.3*mat* – 2017 |
| *S. racemosa* | –4.0*mwmt* + 7.0*cti* – 0.31*cti*^2^ – 0.21*map* + 0.00012*map*^2^ + 109 |
| *R. parviflorus* | 0.35*dd0* – 0.00015*dd0*^2^ – 141*hli* + 112*hli*^2^ + 0.018*ppt_wt* – 5.7*sai* – 1.9*tmn01* – 193 |
| *R. idaeus* | 1.0*mcmt* – 0.050*mcmt*^2^ – 152*hli* + 119*hli*^2^ + 45 |
| *V. myrtillus* | –0.023*cc* – 0.70*cti* – 1.4*dcf* + 0.64*ppt_sm* – 0.0015*ppt_sm*^2^ – 61 |
| *V. caespitosum* | –398*td* + 8.1*td*^2^ – 0.71*cti* + 0.077*pas* – 0.074*cc* – 6.8*dcf* + 4857 |
| *V. scoparium* | –4.6*dcf* + 0.093*msp* – 2.0*hrb* + 157*emt* + 1.9*emt*^2^ + 3292 |
| *F. virginiana* | –3.8*cb* + 0.083*cc* – 0.0013*cc*^2^ – 65*emt* – 0.77*emt*^2^ + 1.1*hrb* – 0.11*cti* + 0.022(*cb* × *cc*) + 0.55(*cti* × *cb*) – 1355.032) |
